# The role of synthetic biology in climate change mitigation

**DOI:** 10.1186/s13062-019-0247-8

**Published:** 2019-08-20

**Authors:** Charles DeLisi

**Affiliations:** Department of Biomedical Engineering, Boston University, 24 Cummington Mall, Boston, MA 02215 USA

## Abstract

**Abstract:**

There is growing agreement that the aim of United Nations Framework Convention on Climate Change, which is to avoid dangerous anthropogenic interference with the climate system, is not likely to be met without inclusion of methods to physically remove atmospheric carbon. A number of approaches have been suggested, but the community appears to be silent on the potential of one of the most revolutionary technologies of the current century, systems and synthetic biology (SSB). The potential of SSB to modulate the fast carbon cycle, and thereby mitigate climate change is in itself enormous, but if the history of genomics is any measure, it is also reasonable to expect sizeable economic returns on any investment. More generally, the approach to climate control has been badly unbalanced. The last three decades have seen intense international attention to emission control, with no parallel plan to test, scale and implement carbon removal technologies, including attention to their economic, legal and ethical implications.

**Reviewers:**

This article was reviewed by Richard Roberts, Aristides Patrinos, and Eugene Koonin, all of whom were nominated by Itai Yanai. For the full reviews, please go to the Reviewers’ comments section.

## Background

For a number of reasons, it should come as little surprise that the various international protocols adopted over the past few decades have not diminished global CO_2_ emissions [1, 2]. Among them are the accessibility, reliability, cost effectiveness and scalability of coal, which weighs heavily in favor of its use by developing nations to meet their growing energy demands; the availability and cost effectiveness of natural gas, especially in the U.S. and Russia; and the absence of market based regulatory strategies (carbon taxes or cap and trade systems), without which the necessary emission reductions are unlikely to be achieved.

## Opinion

The good news is that for the US and other technologically advanced nations, the cost of generating electricity by renewable energy technologies such as wind and solar power is economically competitive with that of coal (https://www.lazard.com/perspective/levelized-cost-of-energy-and-levelized-cost-of-storage-2018/), and the balance will continue to tilt toward carbon-neutral energy sources as they develop and mature. As of now, however, most signatories to the Paris Accord are not meeting their targets [3] and developing countries will take decades to switch to a new primary energy source [4], while they simultaneously struggle to meet their growing energy needs. More generally, a number of economic sectors including agriculture, construction, some forms of transportation, and waste will not be easily impacted by carbon-neutral sources [5]. All of this suggests that absent the physical removal of carbon from the atmosphere (so-called negative emission), the planet’s global average temperature at the end of the century is unlikely to be less than 2 °C above its preindustrial level [6].

In fact, numerous carbon dioxide removal (CDR) strategies have been proposed [7, 8], although few have been incorporated into integrated assessment models. Surprisingly, one of the most revolutionary technologies of the current century, synthetic and systems biology (SSB) [9–11] has, with the exception of some brief remarks [12], been virtually absent from the discussion.

SSB, which enables the design and modulation of cellular phenotypes in ways that could scarcely have been imagined even a decade ago, can amplify the power of the entire range of land management practices [13], from agriculture to forestry, that help regulate the flow of carbon between its reservoirs. In principle, the development of land management technologies using the modern methods of molecular science [14] can not only remove atmospheric carbon, but will, as with the Human Genome Project [15], likely lead to multiple insights and opportunities having wide- ranging scientific and economic ramifications well beyond climate control.

**Fig. 1:**
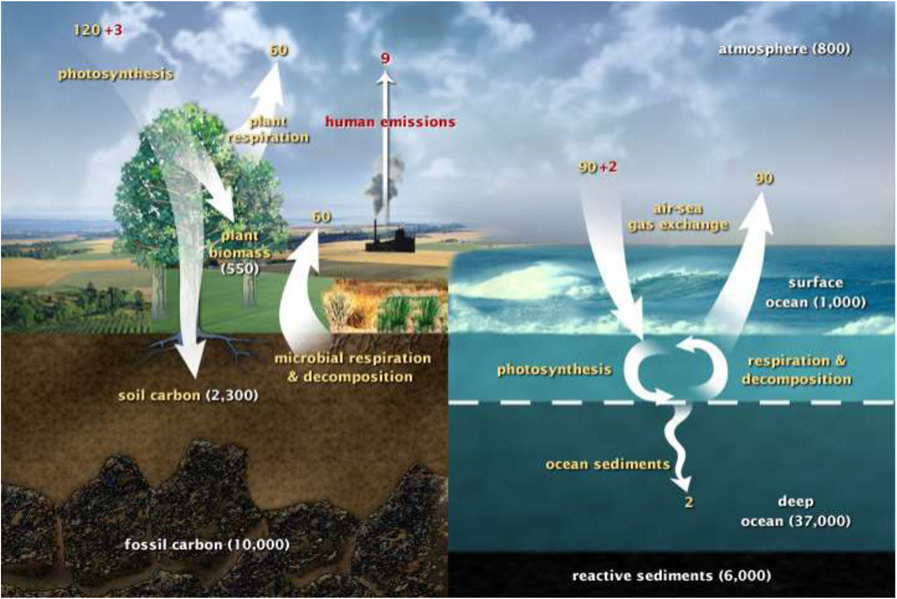
The fast carbon cycle (numbers are approximate). In the steady state (no anthropogenic sources) 120 of the 800 GtC of atmospheric carbon is absorbed and released by terrestrial life during annual cycles of growth and decay. http://earthobservatory.nasa.gov/Features/CarbonCycle/adapted.fromU.S.DOE,Biological.and.Environmental.Research.Information.System

SSB offers the possibility of modulating the fast carbon cycle, the continuous exchange of carbon between atmosphere, land and sea on a decadal time scale. As summarized in Fig. 1, every year some 120 gigatons of carbon (GtC) are removed from the atmosphere by terrestrial photosynthesis, and every year essentially the same amount is returned by plant and microbial respiration. Even a small reduction in the return step can substantially reduce atmospheric carbon.

Many scientific pathways are potentially available for vastly accelerating the science of land management via SSB. These include development of engineered plants with increased root to shoot ratio; amplification of processes controlling soil mineral absorption and mineral carbonation (e.g. calcium oxalates by cacti; silicon phytoliths by multiple plant species); engineered soil microbes that would convert organic carbon into stable carbonates, and pigment modification of selected tree species to increase albedo.

Equally importantly, new methods to engineer plants with reduced requirements for fertilizers, increased yields, more efficient machinery for nitrogen fixation and photosynthesis [16], and reduced need for water, can enable the use of currently non-arable land for plant growth. Such technologies could, in addition, help maintain a stable supply of crops as the planet warms and conditions for vegetative growth change. They also open a potentially important synergism with the CDR strategy that has so far received the greatest attention--bioenergy with carbon capture and storage, which requires vast tracts of arable land.

Some feeling for the potential power of carbon cycle modulation can be illustrated by back of the envelope calculations of CDR^1^ if trees could be engineered to convert a fraction of the carbon that is ordinarily respired into, for example, stable carbonates.

**Fig. 2:**
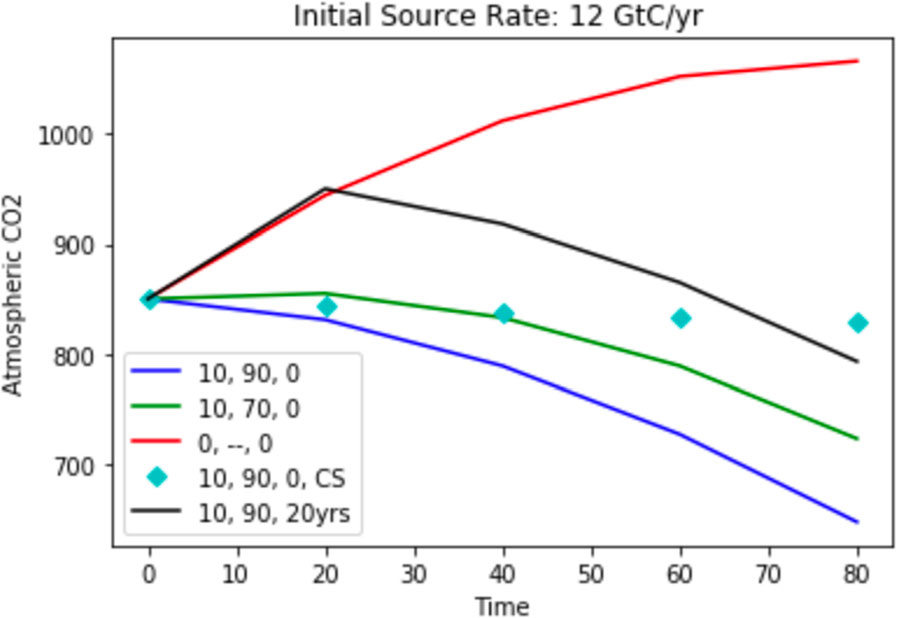
Atmospheric carbon dioxide as a function of time in years. The triplets in the legend represent the percentage of engineered trees, their efficiency, and the mitigation start time. The last entry (black) shows the course of atmospheric CO2 when mitigation is delayed 20 years. CS indicates that the source remains constant at 12 GtC/year; in all other cases it decreases uniformly to zero over an 80-year period

The impact of emission rate control, and the effect of delayed implementation (Fig. 2) are especially worth noting. With respect to the former, after 60 years with 10% of the planet’s trees engineered to operate at 70% (90%) efficiency, the atmospheric CO2 level would drop from 850 GtC to 789 (727) GtC (green and blue lines, respective) provided the emission rate drops uniformly to zero over an 80-year period. If the emission rate is not reduced, but remains at the initial value of 12 GtC/year (cyan diamonds), atmospheric CO2 is reduced very little: from 850 GTC to 833 GtC, for 90% efficiency with 10% of the trees engineered. On the other hand, if the emission rate is reduced to 0 without carbon capturing trees (red), the CO2 level increases by 197 GtC, to 1047 GtC, giving a net difference of 214 GtC. These last two results emphasize the importance of a strategy that combines emission control with CDR.

With respect to delayed implementation, atmospheric CO2 is currently increasing at approximately 5 GtC/year, so a 20-year delay would add approximately 100 GtC to the atmosphere. Remediation would then take the CO2 level from 950 GtC to 793 GtC (black), again with 10% of the trees working at 90% efficiency. In other words, the atmospheric carbon 80 years from now would be 793 GtC. This should be compared to 647 GtC (blue), the atmospheric concentration 80 years from now if remediation were to begin today.

Trees were used to fix ideas because they are the dominant engine of carbon turnover, so in the longer term I expect they will be the major mitigant. On the other hand, the complexity of their genomes will no doubt delay implementation of a CDR strategy, especially relative to a potentially quick start using other plant genomes that may be more readily engineered. Although I wish to avoid being overly detailed and prescriptive about strategy at this initial phase of discussion, I note that the model on which Fig. 2 is general and can be used to compare different plant species, provided one knows something about their contributions to the carbon cycle. What is needed most at this point is a delineation and careful vetting by the community of various options, their time scales for implementation and their effectiveness.

Finally, the primary objective of this article is not to advocate SSB in preference to other negative emissions technologies: not enough is known either about which of the many possible SSB interventions can be implemented in the short term, or about their environmental impacts. But it is, in my opinion, a mistake to omit SSB from serious consideration. More generally, unlike three decades of intense international attention to emission control, there has been no parallel effort to develop a plan for testing, scaling and implementing negative emission technologies, including attention to their economic, legal and ethical implications. There is precedent for eminently successful large-scale engineering projects in times of emergency (the Manhattan Project) or when opportunities exist for extraordinary advances in human knowledge (the Moon-shot and the Human Genome Project). Addressing the world’s climate offers both.

## Reviewers’ comments

### Reviewer’s report 1

Richard Roberts, New England Biolabs (Nominated by Itai Yanai)

This is a timely article that deals with an aspect of climate change that is rarely discussed in a realistic sense, namely carbon capture by bio-engineering plants. In general, previous discussions have focused on the relative impractical nature of employing carbon-capture technologies on a scale sufficiently large to have any significant impact on the amounts of CO2 and methane in the atmosphere. Using back-of-the-envelope calculations DeLisi attempts to show that biological carbon capture could be a practical solution to mitigating CO2 build-up. I am not equipped to deal fully with the math, which should be left to someone more familiar with the practical aspects of plant metabolism. However, the important take-home message from my perspective is that this is an area worth a much fuller exploration. Trees are probably not the ideal example as most of the trees in the world are in forests that could not easily be manipulated or are sufficiently slow growing that it would take decades for them to reach a point where thy might have a significant impact. However, *agricultural crops grown for food* production might be a better short-term target. *First, many are already understood genetically sufficiently well that further modification might be relatively straightforward*. They are grown in vast amounts and while converting CO2 to carbonate may not be the ideal way of storing the captured carbon other final products such as carbohydrates might be feasible. Second, they are probably a better choice that most trees because *much research would likely be needed to produce trees with the desired properties.* Time would be a big issue as typically tree geneticists start their work but have retired and relied on first- or second-generation students to explore the results of their genetic studies. *With highly polyploid chromosomes manipulating their genomes can be very tricky*. Nevertheless, despite the criticisms above, this is a very useful article that with a small amount of rewriting to mention the practical details of tree biology and introducing *the idea that other species some of which might be food plants might offer more practical advantages*, might have some impact. In light of the current situation as many ideas as possible need to be aired and tested. We have precious little time left to try and resolve the crises that will arise if climate change continues unabated. The last paragraph is particularly timely. *A couple of small points: 1.* Figure 1*. This is a nice figure, but the numbers are a little difficult to read. 2. P3, line 18. Should read “a net difference of” 3.* Figure 2*. The colors are a little difficult to distinguish*. Perhaps, two of the lines could be dotted for clarity.

Author’s response: *Dr. Roberts makes good points, and I’m grateful to him for starting the exchange of ideas. The fact that many agricultural plants are much better understood than trees, and would offer a quicker start is especially important given the urgency of the problem. This point, the importance of a quick start, is reinforced by Fig.* *2**, which illustrates the dramatic effect of a 20-year delay in implementing a CDR strategy. I chose trees only because they are the single largest CO2 absorbers. With respect to policy, the choice of other plants in the near term is, as Dr. Roberts indicates, crucial and remarks bearing on these issues have been added. More generally, with respect to choices, what’s needed now is a careful vetting by the community of the various technical strategies including the state of the underlying science, plausible timelines and tradeoffs between different approaches, costs, environmental impact, efficacy and governance. This is something that we’re beginning to see for other CDR strategies (see comments by Dr. Patrinos) and will, I expect, soon be starting for synthetic biology.*

### Reviewer’s report 2

Aristides Patrinos, Chief Scientist, the Novim Group (Nominated by Itai Yanai).

I applaud the message conveyed by the author in this paper. Despite three decades of intensive research, several in depth international assessments of impacts, and a series of conventions and treaties, action on reducing the emission of greenhouse gases in the atmosphere has been at best lackluster.

As the author emphasizes, there is a movement among the serious-minded to focus attention and eventually significant R&D resources to ways of removing carbon from the atmosphere and safely sequester it for long periods of time. The National Academy of Sciences (NAS) has recently released a report: **Negative Emissions Technologies and Reliable Sequestration: A Research Agenda (2019)**. Several organizations are currently converting the NAS recommendations into action plans and I am currently involved in one such effort.

Although biotechnology is included in the range of approaches to capture carbon from the atmosphere and safely sequester it, there, is in my opinion, insufficient emphasis so far on utilizing the most “cutting-edge” tool that the author of this paper has highlighted, systems and synthetic biology. As he has emphasized, it may very well be the most revolutionary game-changer in terms of efficacy and cost. However, I also agree with the author that such a game-changer will also require a comprehensive effort addressing the ecological effects of this biotechnology as well as its ethical, legal, and societal implications.

A few additional comments on this paper:

*The delays in adopting renewable technologies has been the availability and relatively low cost of natural gas rather than coal. Moreover, another wild card may be nuclear energy. Facing a possible demise in the US, it nevertheless is proliferating in several parts of the world and may end up with a significant role in mitigating climate change.*

*On the policy front, the most important instrument for stabilizing greenhouse gas concentrations in the atmosphere is placing a price on carbon, either through a tax or* via *the purchase of permits to emit it.*

Author’s response: *Dr. Patrinos correctly identifies natural gas as a primary competitor of renewable technologies, especially in the United States and Russia. I also agree that, as far as the world in total is concerned, nuclear energy is not dead as an important energy source -- although I hesitated to mention it for fear of taking the focus off the main thrust of this article*.

Figure 2 illustrates the importance of implementing an effective emission control strategy, but I avoided discussing any specific economic approaches. Dr. Patrinos correctly calls attention, as I now have in the revision, to the importance of a carbon tax, the economics of which has been analyzed in great detail by William Nordhaus, recipient of the 1918 Nobel Prize in economics.

### Reviewer’s report 3

Eugene Koonin, NCBI, NLM, NIH (Nominated by Itai Yanai).

In this Opinion article, Charles DeLisi propose the use of engineered plants, with part of the CO (2) that is normally emitted through respiration redirected to the formation of stable carbonates, for climate mitigation by removing CO (2) from the atmosphere. Rough estimates are presented demonstrating a remarkable efficiency of the proposed approach. More general, the article advocates serious consideration of Systems and Synthetic Biology as a source of climate mitigation strategies.

I found the article to be excellent, succinct, original and clear. My only, and certainly, optional suggestion would be to move the calculations of the efficacy of the proposed strategy of CO (2) removal from a footnote to the main text and explain the assumptions behind these calculations in somewhat greater detail.

Author’s response: *I appreciate Dr. Koonin’s remarks. The technical details are in a footnote so as not to disrupt the flow of the narrative. I have, however, expanded the explanation, and trust that it is now clear.*

## Data Availability

Data generated are included in the article.

## Footnotes

1 In general, the anthropogenic contribution to rate of change of atmospheric carbon, C, due to carbon dioxide consists of a source term S(t) whose form is determined by the requirement of meeting some regulatory target; and a loss term related to a carbon dioxide removal strategy. Going from the general to the particular, the source term is taken to be a linearly decreasing function of time, t, in years; viz, S(t) = R_0_ - δt, where R_0_ is the initial rate of emission in gigatons of carbon (GtC) per year, and δ is determined by the requirement that the emission rate be brought to zero in 80 years. The loss term is proportional to the concentration of atmospheric carbon, and the characteristic time, τ, for its irreversible removal. In particular, τ = (e*T*0.075/yr.)^− 1^, where 0.075/yr. (= 60(GtC/yr) / 850GtC) is the natural fractional turnover rate of carbon by non-engineered trees, and T is the fraction of the planet’s trees that are engineered to remove carbon irreversibly with efficiency, e. In the absence of engineered trees, all carbon absorbed during growing season will on average be returned to the atmosphere during decay, so there would be no net removal (this ignores deep ocean and terrestrial sinks which remove carbon very slowly on the time scale of interest). If all trees are engineered, the average rate of irreversible removal is 0.075GtC/yr, and in general the average removal rate is 1/τ. The differential equation describing the rate of change in atmospheric carbon is then dC/dt = S(t) – C/τ, whose solution is C = exp (τ−t/() [C_0_ – f τR_0_ - fδτ^2^] + fτ[S(t) +τ δ] (t < 80 years). The natural turnover rate is assumed to be invariant, and natural carbon sinks are ignored

## References

[1] Friedlingstein P (2014). Persistent growth of CO_2_ emissions and implications for reaching climate targets. Nat Geosci.

[2] International Energy Agency, Market report series: Coal 2018. Analysis and forecasts to 2023. 2018.

[3] Victor DG (2017). Prove Paris was more than paper promises. Nature.

[4] Marchetti C, Nakicenovic N (1979). The dynamics of energy systems and the logistic substitution model RR-79-13.

[5] IPCC AR5 Climate Change 2014, Mitigation of Climate Change, WG, Energy Use Sectors. 2014.

[6] Sanford T (2014). The climate policy narrative for a dangerously warming world. Nat Clim Chang.

[7] Williamson P (2016). Scrutinize CO_2_ removal methods. Nature.

[8] Minx Jan C, Lamb William F, Callaghan Max W, Fuss Sabine, Hilaire Jérôme, Creutzig Felix, Amann Thorben, Beringer Tim, de Oliveira Garcia Wagner, Hartmann Jens, Khanna Tarun, Lenzi Dominic, Luderer Gunnar, Nemet Gregory F, Rogelj Joeri, Smith Pete, Vicente Vicente Jose Luis, Wilcox Jennifer, del Mar Zamora Dominguez Maria (2018). Negative emissions—Part 1: Research landscape and synthesis. Environmental Research Letters.

[9] Hugh DG, Wright P, Hailstones D (2018). Emerging opportunities for synthetic biology in agriculture. Genes.

[10] Cameron DE, Bashor CJ, Collins JJA (2014). Brief history of synthetic biology. Nat Rev Microbiol.

[11] Church G, Regis E (2012). Regeneisis: how synthetic biology will reinvent nature and ourselves.

[12] Dyson F. Climate and land management. In: A Many-colored Glass: Reflections on the Place of Life in the Universe. Charlottesville: University of Virginia Press; 2007.

[13] Lenton TM (2010). The potential for land-based biological CO_2_ removal to lower future atmospheric CO_2_ concentration. Carbon Management.

[14] Vickers C, Small I. The synthetic biology revolution is now – here's what that means. The Conversationhttps://phys.org/news/2018-09-synthetic-biology-revolution.html. 2018.

[15] DeLisi C (2008). Santa Fe 1986: human genome baby steps. Nature.

[16] Jez J, Soon GL, Sherp A (2016). The next green movement: plant biology for the environment and sustainability. Science.

