# Correction to: The role of synthetic biology in climate change mitigation

**DOI:** 10.1186/s13062-019-0254-9

**Published:** 2019-11-29

**Authors:** Charles DeLisi

**Affiliations:** Department of Biomedical Engineering, Boston University, 24 Cummington Mall, Boston, MA 02215 USA

**Correction to: Biol Direct (2019) 14:14**

**https://doi.org/10.1186/s13062-019-0247-8**

After publication of this article [1], the author brought to our attention that there are some errors in the article.

The first error is in the equation of the footnote 1. The correct equation should be: C = exp(-t/ τ) [C_0_ – f τR_0_ - fδτ^2^ ] + f τ [S(t) + τδ] (t < 80 years), rather than C = exp (τ −t/() [C_0_ – f τR_0_ -fδτ^2^] + f τ [S(t) + τδ] (t < 80 years) in the previous publication.

The second error is in the beginning of the third line from the bottom of the footnote currently reads dC/dt = S(t) - C/ τ whereas it should read dC/dt = f S(t) - C/ τ.

The last error is the year in the last sentence of the response to Reviewer’s report 2: it should be 2018 rather than 1918.
